# C-reactive Protein to Albumin Ratio among Patients Admitted to Intensive Care Unit of a Tertiary Care Hospital: A Descriptive Cross-Sectional Study

**DOI:** 10.31729/jnma.7047

**Published:** 2021-12-31

**Authors:** Pratiksha Gyawali, Himal Shrestha, Vivek Pant, Prabodh Risal, Sharad Gautam

**Affiliations:** 1 Department of Clinical Biochemistry, Kathmandu University School of Medical Sciences, B.P Highway, Panauti, Kavrepalanchok, Nepal; 2 Department of Physiology, Anatomy and Microbiology, La Trobe University, Bundoora, VIC, Australia; 3 Department of Clinical Biochemistry, Samyak Diagnostic Pvt. Ltd, Lalitpur, Nepal; 4 Department of Clincal Biochemistry, Kathmandu University School of Medical Sciences, B.P Highway, Panauti, Kavrepalanchok, Nepal; 5 Department of Laboratory Medicine, Cleveland Clinic Akron General, Akron, Ohio, USA

**Keywords:** *albumin*, *C-reactive protein*, *critical illness*, *sepsis*

## Abstract

**Introduction:**

Sepsis is the most common cause of mortality among patients admitted to intensive care unit. There is emerging evidence on the role of C-reactive protein to albumin ratio (C-reactive protein/Albumin) in predicting outcomes in patients with critical illness and sepsis, admitted to intensive care unit. We aimed to find out the median value of C-reactive protein/Albumin ratio among patients admitted to intensive care unit of a tertiary care hospital.

**Methods:**

We conducted a descriptive cross-sectional study of 110 critically ill patients (>18 years old) admitted to intensive care unit of Dhulikhel Hospital from April, 2014 to June, 2016. The ethical approval (Reference number.51/16) was obtained from Institutional Review Committee at Kathmandu University School of Medical Sciences. C-reactive protein/albumin ratio was calculated from records of patients admitted to the intensive care unit. Convenience sampling was done. Data were entered into Microsoft Excel and analysed using Statistical Package for Social Sciences version 20. Point estimate at 95% Confidence Interval was calculated along with frequencies and percentages for binary data.

**Results:**

Among 110 patients admitted to the intensive care unit, the median value of C-reactive protein/Albumin ratio was found to be 2.5 (Interquartile range: 0.6-4.1). Out of these patients, 44 (39.5%) patients were septic and their median C-reactive protein/albumin ratio was 3.4 (Interquartile range: 3.1-4.5).

**Conclusions:**

Our study showed higher median C-reactive protein/Albumin when compared to other similar studies. Monitoring of C-reactive protein/albumin level in a patient admitted to intensive care unit could be useful for stratifying patients with a high risk of developing sepsis.

## INTRODUCTION

Sepsis is the most common cause of mortality among patients admitted to Intensive Care Unit (ICU).^1,2^ Recent evidence suggests the association of higher C-reactive protein (CRP)/Albumin value with sepsis.^3,4^ On admission CRP/Albumin has been reported to have high predictive capability of sepsis.^5^ Various researches suggest its role as an independent predictor of mortality among patient with sepsis in ICU.^6,7^

Newer biomarkers like procalcitonin-Interleukin-6 can better predict septicemia,^8^ however these are costly and not extensively used. Outcome predicting scoring systems such as Acute Physiology and Chronic Health Evaluation (APACHE) III, Simplified Acute Physiology Score (SAPS) involves multiple variables.^9^ The early distinction of sepsis status in critically ill patients admitted to ICU is important for timely medical management and improving outcome.^8^ CRP/Albumin could be a cost-effective two-variable biomarker to stratify patients with sepsis from non-sepsis.

We aimed to find out the median value of CRP/Albumin ratio among patients admitted to intensive care unit of a tertiary care hospital.

## METHODS

This is a descriptive cross-sectional study of critically ill patients at the ICU of Dhulikhel Hospital in Nepal. This study included clinical and laboratory data of 110 patients over a period of 27 months (April, 2014 to June, 2016) from patients admitted to the ICU. Ethical clearance was obtained from Institutional Review Committee of Kathmandu University School of Medical Sciences (Reference number: 51/16). Convenience sampling was done. As we reviewed patient's record, the criteria for informed consent from the patient was waived by committee. We included patients of age at least 18 years meeting the following criteria: a) CRP and Albumin were measured during the day of ICU admission, b) A complete diagnosis of the underlying cause was documented, c) The outcome of the patient i.e. Died (ICU mortality) and Improved (shifted to the ward or discharged from ICU) was documented.

Patients who were admitted for less than 24hours, required multiple admissions, and transferred to the other hospital were not studied.

The sample size was calculated using the following formula:

n = Z^2^ × σ^2^ / e^2^

  = (1.96)^2^ × (0.5)^2^/ (0.01)^2^

  = 96

Where

n = minimum number of sample required
Z= 1.96 at 95% Confidence Interval (CI)
σ = standard deviation taken for maximum sample size, 0.5
e = margin of error, 1%

The calculated sample size was 96. We have included 110 patients in our study. The clinical data were abstracted from the record file of ICU and the laboratory data were abstracted from the department of clinical biochemistry from laboratory software, Modular Interactive Data Acquisition System (MIDAS) version 3.2, in accordance with patient's hospital registration n umber. Data were collected by on e author (HS) using convenient sampling strategy in a paper based abstractor form, maintaining the anonymity. The data included patient's hospital registration number, age, gender, duration of ICU stay, diagnosis of illness and outcome. Underlying disease conditions during the ICU admission were classified according to the International Classification of Disease (ICD-10).^10^ Value of CRP, and albumin that were measured within twenty-four hours of ICU admission, were collected. The reference range for CRP was 5mg/L to 120mg/L and for albumin was 3.5gm/dL to 5.5gm/dL. CRP/Albumin was calculated by dividing serum CRP level (mg/dL) and serum albumin level (gm/dL).

We entered the data into Microsoft Excel and exported it to Statistical Package for Social Sciences (SPSS) version 20.0. Point estimate at 95% Confidence Interval was calculated along with frequencies, mean and percentages for binary data.

## RESULTS

Among 110 patients admitted to the intensive care unit, the median value of C-reactive protein/Albumin ratio was found to be 2.5 (Interquartile range: 0.6-4.1).

The median age of patients was 45 years (range: 1898). The median duration of ICU stay was 5 days and 38.2% of patients stayed in ICU for more than 5 days (Table 1).

**Table 1:** Baseline characteristics of the patients admitted to the intensive care unit (n=110).

Characteristics	n (%)
**Age (in years)**	
18-45	61 (55.5)
>45	49 (44.5)
**Gender**	
Male	50 (45.5)
Female	60 (54.5)
**Ventilation Status**	
Ventilated	21 (19.1)
Not Ventilated	89 (80.9)
**Duration of ICU stay (in days)**	
1-5	68 (61.8)
>5	42 (38.2)
**Sepsis**	
Present	44 (39.5)
Absent	66 (60.5)
**Outcome**	
Died	28 (24.4)
Improved	82 (75.6)

The minimum duration of ICU stay was 1 day and the maximum was 28 days. Of those who stayed in the ICU, 19.1% of them required mechanical ventilation. Similarly, 24.4% of patients died while 75.6% improved and survived during their ICU stay (Table 2).

**Table 2:** Underlying causes of ICU admission (n = 110).

Disease Category	n (%)
Diseases of the genitourinary system	3 (2.73)
Endocrine, nutritional, and metabolic diseases	2 (1.82)
Certain infectious and parasitic diseases	11 (10)
Diseases of the respiratory system	26 (23.6)
Diseases of the digestive system	31 (28.1)
Pregnancy, childbirth, and the puerperium	9 (8.1)
Diseases of the nervous system	11 (10.0)
Diseases of the circulatory system	9 (8.1)
Neoplasms	2 (1.8)
Injury, poisoning and certain other consequences of external causes	6 (5.4)

The CRP, Albumin and CRP/Albumin ratio among patients with sepsis is shown in Table 3. The median CRP in those with sepsis was 121mg/dl and CRP/Albumin among septic patient was found to be 3.4. (Table 3).

**Table 3:** CRP, Albumin and CRP/Albumin value among critically ill patients in different groups.

	CRP mg/dL	Albumin g/dL	CRP/Albumin
Parameter	Median (25th, 75th percentile)	Median (25th, 75th percentile)	Median (25th, 75th percentile)
No Sepsis	39.5 (9.5, 107.5)	3.2 (2.7, 3.7)	1.4 (0.3, 3.4)
Sepsis	121 (91.5, 121)	3.0 (2.4, 3.4)	3.4 (3.1, 4.5)
Survived	91 (21.3, 121)	3.2 (2.7, 3.7)	2.6 (0.52, 4.1)
Didn’t Survive	80.5 (24, 121)	2.8 (2.3, 3.2)	3.2 (0.73, 4.3)

## DISCUSSION

In this study review we aimed to find out the median value of the CRP/Albumin ratio among patients admitted to intensive care unit. Our study showed higher median CRP/Albumin. The results agree with the finding of other study that reported high CRP/Albumin among patients.^22^ Sepsis was diagnosed in 40% of the patients admitted to ICU. The diseases associated with the digestive and respiratory system were the most common underlying cause for ICU admission. On admission, CRP and CRP/Albumin ratio were higher among patients admitted to ICU with sepsis.

Worldwide assessment of the burden of critical illness showed that sepsis is the most common cause of mortality and morbidity among patients admitted to ICU.^2^ An audit of ICUs from 84 countries revealed a 29.5% prevalence of sepsis of which the highest prevalence of sepsis was in South East Asia.^1^ We found that 74.5% of patients admitted to ICU improved, whereas 25.45% died and 18.18% required ventilation. The patient with sepsis commonly requires mechanical ventilation, around 68% of patients identified with sepsis received mechanical ventilation than the non-sepsis (38.8%) patients worldwide.^1^ In addition, patients diagnosed with sepsis has a higher ICU mortality rate and length of stay than non-sepsis.^1^ These findings suggest that sepsis is a common complication in ICU and patient admitted to ICU should be prioritized for early identification of sepsis to improve outcomes through better management.

Diseases of the digestive system and the respiratory system were common underlying causes for ICU admission in our setting. The respiratory tract followed by the abdomen has been reported as the common source of sepsis in ICU in worldwide assessment.^1^ Thus, underlying causes that involve respiratory and abdominal organs needs increased vigilance in a patient admitted to ICU.

Our study showed high median CRP and CRP/Albumin among patients admitted to ICU. CRP, is an acute phase protein synthesized by hepatocytes in response to Interleukin (IL)-6 and is elevated and correlates with inflammation.^11^ A higher value of CRP has been found in patients with sepsis than non-sepsis.^12,8^ CRP has a role as an independent prognostic indicator of sepsis in patients admitted to ICU.^12,13^ A previous study conducted in the ICU of Nepal reports CRP measured within 48 hours of admission as a more sensitive marker of sepsis.^14^ Likewise, Devran, et al.^15^ reported third day CRP value as a lone predictor of mortality in patients admitted to ICU with severe sepsis. Serial monitoring of CRP has been useful in monitoring of recovery from sepsis.^7^ Although there are numerous evidences of CRP as a valuable marker in sepsis, however inconsistency remains in those studies in terms of the time span of data collection. Moreover, CRP has been found to have a moderate role in sepsis diagnosis and procalcitonin has higher diagnostic accuracy than CRP.^16^

We found lower median albumin level among patient diagnosed with sepsis than non-sepsis. Hypoalbuminemia is a common manifestation in critically ill patients^17^ and an independent predictor of outcome in patients admitted to ICU.^18^ Reduced albumin synthesis is seen in acute phase reaction under the influence of IL-6 and Tumour Necrosis Factor (TNF).^19^ A study reported lower albumin value during admission among non-survivors than survivors-but poorly differentiated the two groups.^20^ Likewise-the same study reported lower sensitivity and specificity of albumin to predict hospital mortality.^20^ In addition-albumin level after 24 hour of admission to ICU has been found to predict outcome rather than on admission albumin level.^9^

CRP and albumin show inverse relations during the inflammation.^21^ Similarly-another study found higher ratio of CRP/albumin in patient with septic shock than sepsis alone.^4^ High CRP/Albumin value has been reported in patients with sepsis than non-sepsis in those admitted to ICU with burn injury.^5^

We found higher CRP/Albumin among non-survivor than survivor. Other studies have reported higher CRP/Albumin among non-survivors.^3^ A significant high CRP albumin value of 8.9 [5.1-14.6] and 3.2 [1.6-5.5] was found on admission and discharge among non-survivor septic patients.^7^ Our study did not investigate the role of CRP/Albumin in predicting outcome. However-it has been found that CRP/Albumin value >5.09 predicts survival among patient with sepsis and septic shock with best sensitivity and specificity.^6^ Similarly-CRP/Albumin as better prognostic indicator compared to CRP alone in critically ill.^23^ Several questions remains unanswered at present and future studies on role of CRP to albumin as diagnostic and prognostic marker in sepsis is recommended.

The findings of our study have to be seen in the light of several limitations. The single centered-descriptive cross-sectional nature of the study with a smaller sample size makes the valid generalization of the research finding difficult. Because the data were collected retrospectively, there was insufficient information on criteria for documentation. It is suggested that the association of these factors is further investigated in future studies including appropriate sample size and taking into account established predictive indices.

## CONCLUSIONS

Our study showed higher median C-reactive protein/Albumin when compared to other similar studies. CRP/Albumin is found to be high in patients admitted to intensive care unit-particularly septic patients and could have potential to stratify patients at higher risk of developing sepsis and predicting outcomes of critically ill patients in an ICU setting which warrants the need for higher studies in order to establish association in our setting.
